# A Marked Response to Immunosuppressive Intervention for Abruptly Occurring Cardiac Complications in a Case of Juvenile Systemic Sclerosis Overlapped with Dermatomyositis

**DOI:** 10.1155/2017/1479012

**Published:** 2017-02-21

**Authors:** Tsunehisa Nagamori, Yoichiro Yoshida, Hironori Takahashi, Hideharu Oka, Aya Kajihama, Koichi Nakau, Masaya Sugimoto, Masako Minami-Hori, Hiroshi Azuma

**Affiliations:** ^1^Department of Pediatrics, Asahikawa Medical University, 2-1-1 Midorigaoka Higashi, Asahikawa, Hokkaido, Japan; ^2^Department of Dermatology, Asahikawa Medical University, 2-1-1 Midorigaoka Higashi, Asahikawa, Hokkaido, Japan

## Abstract

Juvenile-onset systemic sclerosis (jSSc) is a rare condition, having unique characteristic features compared to adult-onset SSc. Although cardiac involvement (CI) is known as a leading cause of mortality overall in SSc, the importance of CI in jSSc has not been emphasized. Here we present a 13-year-old female with jSSc overlapped with dermatomyositis (DM) complicated CI. She developed skin thickness and induration, Raynaud's phenomenon, digital pitting scars in fingertips, and skeletal myositis. Oral prednisolone and pulse methotrexate treatment led to the improvement of skin findings; however two weeks after the initiation she suddenly presented with muscle pain and dyspnea within a few days. Cardiac investigations then showed pericardiac effusion and diastolic dysfunction due to significant biventricular hypertrophy causing heart failure. As pericardiac effusion and exacerbation of skeletal myositis were evident, steroid pulse therapy was initiated. Unexpectedly, not only the myositis but also the CI including diastolic dysfunction was improved. She thereafter followed a favorable clinical course without reactivation of the CI or cardiac fibrosis. As a conclusion, close attention to CI must be paid in jSSc patients, especially when skeletal muscle involvement is evident and immunosuppressive therapy may be effective for CI in jSSc in cases where it occurs abruptly.

## 1. Introduction

Systemic sclerosis (SSc) is a generalized connective tissue disease characterized by fibrosis of the skin and internal organs [1]. Among the various internal organs, cardiac involvement (CI) has received a good deal of attention in recent years and is recognized as one of the major causes of mortality in patients with SSc [1, 2]. A broad range of clinical manifestations have been reported to be related to CI in SSc, including myocarditis, myocardial fibrosis, left ventricular systolic and diastolic dysfunction, myocardial ischemia, pericarditis, and arrhythmia [3]. These may be a direct consequence of SSc, the so-called primary CI, or develop secondary to the involvement of other organs, such as lung fibrosis and pulmonary arterial hypertension (PAH) [4].

Although juvenile-onset SSc (jSSc) is uncommon, a series of analyses has shown that it presents unique characteristic features compared to adult-onset SSc, such as a high prevalence of patients developing diffuse cutaneous skin sclerosis subtype (dcSSc) and a high proportion of patients showing skeletal muscle involvement, which often fulfill the criteria of overlap syndrome with dermatomyositis/polymyositis (DM/PM) [5, 6].

The treatment strategy for CI is not well established; however, palliative treatment using vasodilators, such as angiotensin receptor blockers (ARB) and Ca channel blockers, is available [7], while several reports have demonstrated the resolution of CI through the use of immunosuppressive interventions [8]. Herein, we report a 13-year-old patient with SSc with inflammatory myopathy who abruptly developed primary CI, consistent with acute myocarditis, and who was successfully treated by immunosuppressive intervention including methylprednisolone pulse therapy.

## 2. Case Presentation

**Figure 1:**
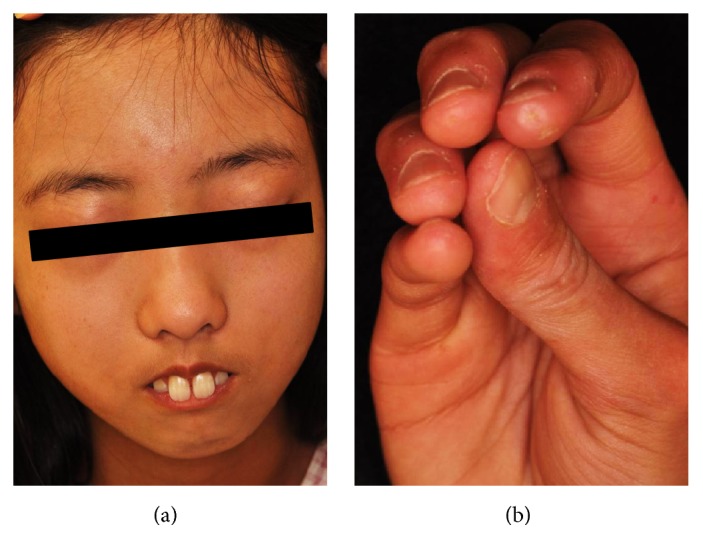
(a) A mask-like face and (b) digital pitting scarring on the right index finger.

**Figure 2:**
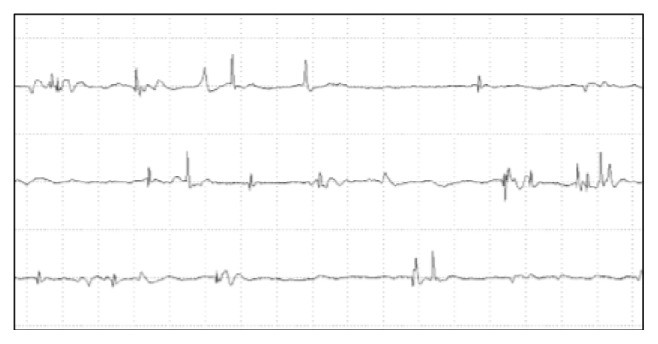
Needle electromyography of the biceps brachii muscle. Polyphasic, short duration, and low-amplitude patterns were observed during voluntary contraction.

**Figure 3:**
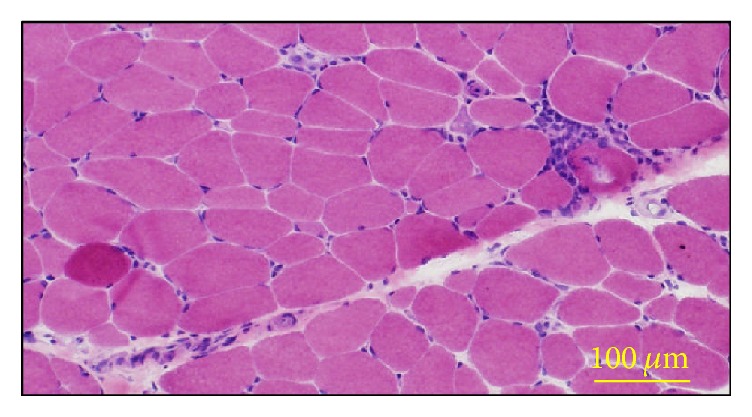
Hematoxylin-Eosin (H&E) staining of the biceps brachii muscle. Muscle fiber necrosis and regeneration and mild inflammatory cell infiltration in the perivascular region of perimysium can be observed.

A 13-year-old female was admitted to our hospital with a one-year history of Raynaud's phenomenon, finger skin thickness, and induration followed by one-month history of digital pitting scars on right index finger. She did not have a past history or family history of rheumatic or other autoimmune diseases. On examination, although her vital signs were normal, a mask-like face (Figure 1(a)) and moderate thickening and induration of skin on the fingers, hands, forearms, feet and lower legs were observed, and her modified Rodnan skin score was 23 [9]. Digital pitting scarring was observed on the right index finger (Figure 1(b)). She showed erythema on the extensor surfaces of the knee and elbow which resembled that in dermatomyositis, although not presented with any significant heliotrope rush, muscle weakness, or muscle pain. As shown in Table 1, laboratory examinations showed mild elevation of serum creatine kinase (CK) and aldolase levels to 648 U/L and 16.8 U/L, respectively. There was no increase in C-reactive protein, erythrocyte segmentation rate (ESR), or other indicators of inflammation. The serum level of N-terminal pro b-type natriuretic peptide (NT-proBNP) or KL-6 was within the normal range. The levels of autoantibodies listed in Table 1 were all normal or negative as far as we examined. Magnetic resonance imaging (MRI) of the lower legs did not show any significant skeletal muscle inflammation; however, needle electromyography revealed myopathic changes (Figure 2). Further, muscle biopsy of the triceps brachii revealed mild perivascular inflammatory cells infiltration, muscle fiber necrosis, and regeneration (Figure 3). On the basis of these results, we diagnosed her as juvenile systemic sclerosis fulfilling The Pediatric Rheumatology European Society (PRES)/American College of Rheumatology (ACR)/The European League Against Rheumatism (EULAR) criteria [10], overlapped with DM, also fulfilling Bohan and Peter criteria [11]. Internal organ complications were carefully investigated; however she had neither esophageal symptoms, nor lung fibrosis in chest computed tomography; also respiratory function was normal in spirometry. We intensely evaluated her cardiac echograph and found it both morphologically and functionally normal, while her electrocardiogram did not show any abnormality.

**Figure 4:**
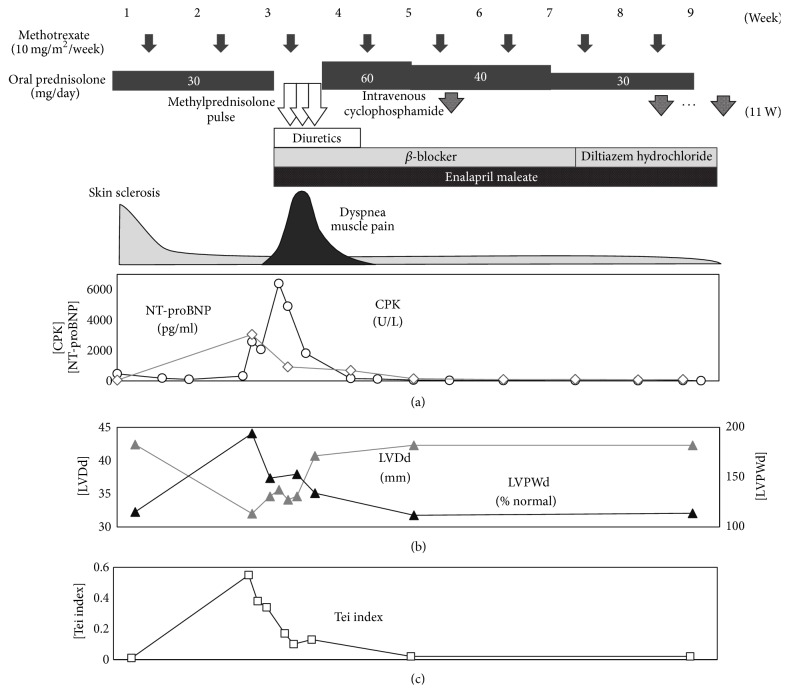
Nine-week time course after the initiation of treatment. Therapeutic agents, symptoms, creatine kinase (CK, shown as open black circles in graph (a)), N-terminal pro b-type natriuretic peptide (NT-proBNP, shown as open gray circles in graph (a)), left ventricular posterior wall end diastole, percent of normal (LVPWD% N, shown as closed black triangles in graph (b)) and left ventricular diastolic diameter (LVDd, shown as closed gray triangles in graph (b)), and Tei index showing left ventricular diastolic and systolic function, calculated as [isovolumic contraction time + isovolumic relaxation time]/[left ventricular ejection time] (Tei index, shown as open squares in graph (c)) are shown. Cardiac dysfunction and exacerbation of myositis occurred abruptly and were resolved after methylprednisolone pulse therapy in combination with palliative therapies for heart failure.

**Figure 5:**
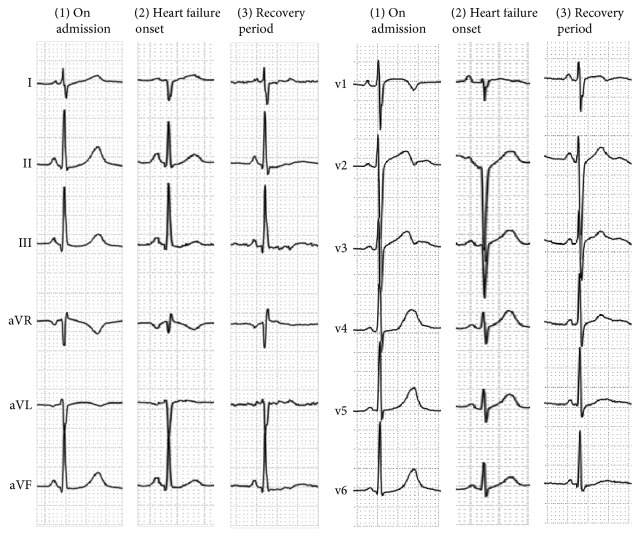
Time course of 12-lead electrocardiogram tracings; (1) at the time of admission; (2) at the onset of heart failure; and (3) during the recovery period (4 weeks after the onset of heart failure). A right axis shift, widespread low voltage, and left ventricular hypertrophy were observed at the onset of heart failure and subsequently resolved.

**Figure 6:**
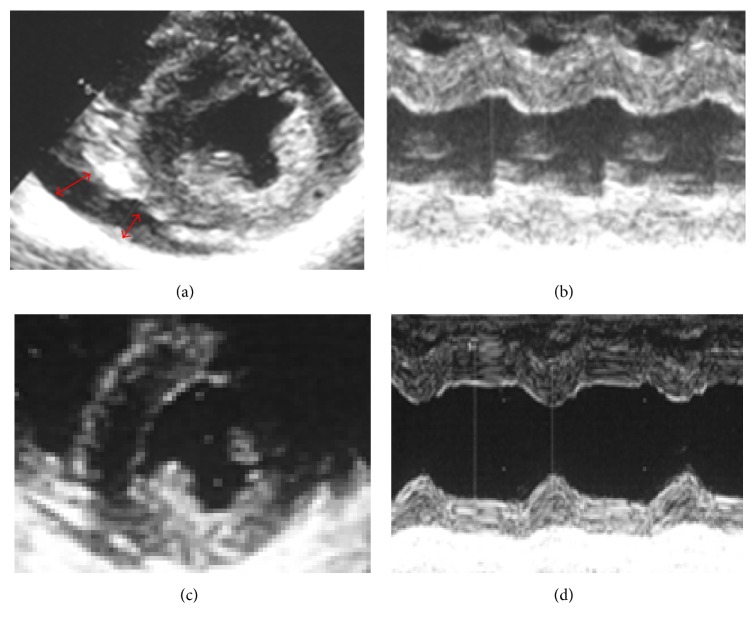
(a, b) Parasternal echocardiography at the onset of heart failure. (a) Short axis view showing pericardiac effusion (red arrow) and all-round ventricular hypertrophy. (b) Motion (M) mode through the left ventricle. The LVDd was 35 mm, left ventricular posterior wall systole (LVDs) 27.6 mm, LVPWD 12.6 mm (196% of normal), and interventricular septal end diastole (IVSd) 13.3 mm (211% of normal). Also, the calculated LVEF was 55%. These findings suggest that diastolic dysfunction due to biventricular hypertrophy resulted in heart failure. (c, d) Parasternal echocardiography 7 days after the initiation of steroid pulse therapy. The LVDd was 40 mm, left ventricular posterior wall systole (LVDs) 23 mm, LVPWD 8.7 mm (133% of normal), and interventricular septal end diastole (IVSd) 10 mm (158% of normal). Also, the calculated LVEF was 80%. Biventricular hypertrophy was improving although still residual, and cardiac contractility was significantly improved.

Initial treatment was started with oral prednisolone (PSL) 1 mg/kg/day (30 mg/day) and methotrexate (MTX) 10 mg/m^2^/week (Figure 4). A significant decrease in myogenic enzyme levels and improvements in skin induration, thickness, and digital pitting scarring in fingertips were observed; however, the patient suddenly complained of general fatigue and spontaneous muscle pain from 12 days after the initiation of treatment. She developed significant exertional dyspnea and preshock status over the following two days. Her electrocardiogram (ECG) showed a right axis shift and widespread low voltage (Figure 5). A subsequent cardiac echocardiography revealed pericardial effusion, not as massive as inducing tamponade, but significant biventricular hypertrophy. The mitral inflow pattern showed diastolic dysfunction resulting in reduced cardiac output (Figure 6). Tricuspid regurgitation, indicative for pulmonary hypertension, was not observed. Serum CK showed an abrupt increase to 6000 IU/L with MM isoform predominance. Her serum N-terminal pro-brain natriuretic peptide (NT-proBNP) level was also increased to 3000 pg/ml. There was no evidence of pulmonary involvement or progressive renal failure. Therefore, palliative treatment for heart failure due to diastolic dysfunction was begun with diuretics (furosemide, 0.5 mg/kg/day), a *β*-blocker (bisoprolol fumarate, 0.625 mg/day), and an ACE inhibitor (enalapril, 2.5 mg/day). In addition, as pericardiac effusion was observed and there was an exacerbation of some form of myopathy, presumably myositis, we immediately carried out steroid pulse therapy (methylprednisolone 30 mg/kg for 3 days). The patient's clinical symptoms improved markedly within one week and her serum myogenic enzyme and NT-proBNP levels were normalized. Surprisingly, not only had her pericardial effusion disappeared, but also myocardial performance significantly recovered within a further two to three weeks (Figure 4). Because steroid pulse therapy appeared to be effective, we assumed her CI as carditis, somehow comprised of inflammatory pathophysiology. Based on this observation, three courses of cyclophosphamide pulse therapy (500 mg/m^2^/dose, once in four weeks) were also carried out along with PSL dose reduction. She showed a favorable clinical course without relapse under the administration of 5 mg of prednisolone, weekly MTX, ACE inhibitor. The endothelin receptor antagonist, bosentan (125 mg/day), was also administrated for secondary prevention of digital pitting scarring. Cardiac investigations including single-photon-emission computed tomography (SPECT) and MRI as well as echocardiography and electrocardiograms were performed repeatedly; however, no further cardiac dysfunction, arrhythmia, or significant findings of focal fibrosis have been observed to date.

## 3. Discussion

Although it is only an anecdotal case report, our observations appear to have certain implications for the management of jSSc and associated CI. An international meta-analysis of 1645 cases demonstrated that overall CI (defined by major conduction disturbances, ventricular arrhythmia, heart failure, or persistent pericardiac effusion) significantly increased the risk of mortality in SSc (hazard ratio 2.8; 95% CI 2.1–3.8) [2]. The prevalence of CI in SSc varies widely according to the definition and the sensitivity of the diagnostic tools employed, as definite certain proportion of cases of CI are insidious, even clinically occult, at the onset [12]. It has been reported that primary CI is more prevalent in SSc patients with a diffused cutaneous subtype (dcSSc) than in limited form [13, 14], skeletal muscle inflammatory myopathy [15, 16], and anti-topoisomerase-I antibody-positive patients [17, 18].

On the other hand, juvenile-onset SSc is a rare condition and the clarification of its overall clinical features is still in progress. Two early multinational surveys revealed that the characteristics of juvenile-onset SSc differ from those of adult SSc, showing a high prevalence of diffuse skin involvement, which is correlated with a very low prevalence of anti-centromere antibodies. Further, skeletal muscle involvement was also reported to be more frequent, even often fulfilling the criteria of DM/PM therefore diagnosed as overlapped syndrome [5, 6]. The long-term outcome of jSSc is thought to be generally favorable due to a lower incidence of internal organ involvement, such as renal involvement, lung fibrosis, or pulmonary arterial hypertension, compared to adult-onset SSc. However, the most common cause of death in jSSc patients is heart failure [5, 6, 19, 20]. Although it has not evident statistically, it may be possible that jSSc is prone to CI, which corresponds with the high prevalence of dcSSc and skeletal myositis known to associate with CI. Careful attention should therefore be paid to the potential development of CI in patients with jSSc.

The pathophysiology of CI in association with SSc remains controversial. A plausible hypothesis for SSc advocates that the three cardinal features of inflammation due to autoimmunity, hyperreactivity of the microvessels, and excessive fibrosis combine to give rise to the disease [21]. As for CI, it has been well studied in association with the abnormal reactivity of microvessels in the myocardium and repeated focal ischemia leading to irreversible fibrosis, and histological examinations showed diffuse patchy fibrosis with band necrosis [22, 23]. On the other hand, other factors such as myocarditis have also been suggested. Pieroni et al. [8] performed endomyocardial biopsies on selected patients with SSc complicated with recent-onset CI and demonstrated the presence of myocarditis in all of these patients. Therefore, as with SSc itself, the pathophysiology of primary CI may be due to a heterogeneous complex of these features.

Our patient, who suffered from jSSc overlapped with DM at the onset, abruptly presented with CI complications. The clinical features of her CI, consisting of abrupt onset, pericardiac effusion, and exacerbation of skeletal myositis, were more indicative of myocarditis, although this assessment had limitation since we have not excluded viral myocarditis or carried out endomyocardial biopsy. However still there was implication that immune suppressive intervention, including glucocorticoid pulse therapy, in addition to palliative therapy against heart failure with diuretics, a *β*-blocker, and an ACE inhibitor, markedly improved her CI as well as the skeletal myositis. Based on that observation, we also carried out cyclophosphamide pulse therapy, which already had evidence for the effect in lung diseases in SSc [21, 24] Several high-sensitive imaging modalities showed no evidence of myocardial fibrosis. A similar case report also suggested that successful outcomes could be obtained with aggressive immunosuppressive treatment [25]. However, generally CI in SSc was described to develop and progress slowly over a number of years even though myocarditis proceeded to heart failure [4]. Therefore such an abrupt illness should be considered as rather an exception than a rule.

We should note that SSc complicated by CI has a heterogeneous pathophysiology and, in addition to the current recommendations, it is possible that aggressive immunosuppressive interventions can be effective for ventricular hypertrophic diastolic dysfunction in cases with acute and recent onset of cardiac manifestations.

## Figures and Tables

**Table 1:** Results of laboratory examinations at the time of admission.

		(Normal)
WBC	6000 (*μ*l)	(3300–8800)
Neutrophil	46.7 (%)	(49–72)
Lymphocyte	47.0 (%)	(24–38)
Monocyte	3.7 (%)	(1.7–8.7)
RBC	455 (10^4^/*μ*L)	(386–492)
Hb	13.0 (g/dl)	(11.6–14.8)
Ht	37.9 (%)	(35.1–44.4)
Plt	22.6 (10^4^/*μ*L)	(15.8–34.8)
PT	13.1 (sec)	(9–13)
APTT	36.6 (sec)	(23–40)
FIB	195 (mg/dl)	(150–400)
D-dimer	<0.5 (*μ*g/ml)	(<1.0)
TP	6.3 (mg/dl)	(6.6–8.1)
ALB	4.3 (mg/dl)	(4.1–5.1)
Tbil	0.4 (mg/dl)	(0.4–1.5)
TC	154 (mg/dl)	(142–248)
TG	139 (mg/dl)	(30–117)
ALP	372 (U/L)	(106–322)
AST	34 (U/L)	(13–30)
ALT	20 (U/L)	(7–23)
LDH	271 (U/L)	(124–222)
*γ*GTP	9 (U/L)	(9–32)
CK	648 (U/L)	(41–153)
MM	93 (%)	(95.8–100)
MB	6 (%)	(0–1.8)
BB	1 (%)	(0–2.7)
ALD	16.8 (U/L)	(6–11)
BUN	10.1 (mg/dl)	(8–20)
Cre	0.33 (mg/dl)	(0.46–0.79)
Na	141 (mEq/L)	(138–145)
K	4.1 (mEq/L)	(3.6–4.8)
Cl	105 (mEq/L)	(101–108)
Ferritin	46.4 (ng/ml)	(5–157)
CRP	<0.10 (mg/L)	(<0.14)
ESR	3 (mm/h)	(3–15)
IgG	1001.2 (mg/dl)	(861–1747)
IgA	57.1 (mg/dl)	(93–393)
IgM	151 (mg/dl)	(50–269)
NT-proBNP	50 (pg/ml)	(29–124)

Antinuclear antibody		(—)
Anti-dsDNA antibody		(—)
Anti-Ro/SSA antibody		(—)
Anti-La/SSB antibody		(—)
Anti-Sm antibody		(—)
Anti-CCP antibody		(—)
Anti-PM-Scl antibody		(—)
Anti-topoisomerase I antibody		(—)
Anti-RNA polymerase III antibody		(—)
Anti-centromere antibody		(—)
Anti-Jo-1 antibody		(—)
Anti-ARS antibody		(—)
Anti-Ku antibody		(—)
Anti-U1RNP antibody		(—)
